# Vertical oxide thin-film transistor with interfacial oxidation

**DOI:** 10.1038/s41598-022-07052-3

**Published:** 2022-02-23

**Authors:** Yeong Jo Baek, In Hye Kang, Sang Ho Hwang, Ye Lin Han, Min Su Kang, Seok Jun Kang, Seo Gwon Kim, Jae Geun Woo, Eun Seong Yu, Byung Seong Bae

**Affiliations:** School of Electronics and Display Engineering, Hoseo University, Asan, Chungnam 31499 Republic of Korea

**Keywords:** Engineering, Materials science

## Abstract

A vertical oxide thin-film transistor was developed with interfacial oxidation for low voltage operation. The gate metal was used as a spacer for the definition of the transistor’s channel as well as the gate electrode. After definition of the vertical side wall, an IGZO (In-Ga-Zn Oxide) layer was deposited, followed by the interfacial oxidation to form a thin gate insulator. Ta was used for the gate material due to the low Gibbs free energy and high dielectric constant of tantalum oxide. A 15 nm tantalum oxide layer was obtained by the interfacial oxidation of Ta at 400 °C under oxygen atmosphere. The thin gate oxide made it possible to operate the transistor under 1 V. The low operation voltage enables low power consumption, which is essential for mobile application.

## Introduction

Thin-film transistors (TFTs) have been being used widely in display backplanes and studied for applications such as wearable displays, stretchable displays and sensing devices^1–10^. Amorphous silicon TFTs were applied to liquid crystal displays (LCDs), and low-temperature polycrystalline silicon TFTs were adopted in high-resolution LCDs and organic light emitting diode (OLED) displays. Recently, indium gallium zinc oxide (IGZO) TFTs have drawn much attention and have been applied to OLED TVs due to their low off-current, higher mobility than amorphous silicon TFTs, and lower process cost than polycrystalline silicon TFT^11^.

Low power consumption is necessary for more extended applications such as internet of things (IoT) and any mobile applications. Low operation voltage is essential for low power operation because the power is inversely proportional to the square of the operation voltage. Since a TFT has a metal–insulator–metal (MIS) structure, effective ways to reduce the operation voltage are decreasing the thickness of the gate insulator and using a high dielectric-constant material. Both the decrease of the thickness and increase of the dielectric constant increase the induced charge in the channel by the gate voltage, which results in a decrease of the threshold voltage of the TFT and enables the low voltage operation of the TFT.

The drain voltage should also be decreased for small operation voltage of a TFT. Since a short channel length increases the electric field between the source and drain, a decrease of the channel length enables the decrease of the drain voltage without the decrease of the on-current. Low-voltage IGZO TFTs were reported using thin and high dielectric gate insulators^6–9^. For the thin gate insulator, anodic oxidation of aluminum was used, and a 1 V IGZO TFT was achieved^6^. A high dielectric constant was also a reason for a low threshold voltage as well as thin gate insulator. A high-dielectric-material such as Ta_2_O_5_ was also applied for the low voltage operation of an IGZO TFT^7^. Sub-10 nm thin SiO_2_ was also obtained by plasma-enhanced chemical vapor deposition (PECVD) for the low voltage operation of an IGZO TFT^9^.

In this study, we developed a 0.5 V TFT without a deposition process for the gate insulator. In a vertical TFT, usual way to form gate insulator is vacuum deposition such as CVD, PECVD, ALD (atomic layer deposition) and so on. In this study, interfacial oxidation between a gate metal and an IGZO layer was used instead of vacuum deposition for the gate insulator, which is the first try to the oxide vertical TFT and remove the deposition process and reduce plasma induced deterioration of the layer during plasma process.

Interfacial oxidation between metal and IGZO layers was observed in the source drain contact of the IGZO TFT. In oxide TFTs, source/drain electrodes contact the IGZO layer, which invokes a reaction between the metal and active oxide layer. Oxidation reactions between the source/drain metals (such as Al and Ti) and an active oxide layer such as IGZO were reported to degrade TFT characteristics by the increase of the source/drain contact resistance^12–14^. However, in this study, interfacial oxidation was applied to the interface between the gate metal and the IGZO active layer for the gate insulator.

Since the electrical properties of the IGZO are very sensitive to defects and vacancies as well as impurities such as hydrogen and water vapor, the plasma process which generates energetic ions degrade the electrical properties of the IGZO TFT. Interfacial oxidation does not use plasma, so there are no plasma-induced defects after forming the gate insulator on the IGZO layer. Moreover, a very thin gate insulator around several tens of nanometers thick can be easily obtained by interfacial oxidation.

To reduce the source/drain voltage, a vertical channel TFT (VTFT) was adopted for short channel length. In a VTFT, a short submicron channel can be realized easily because the channel length is defined by the thickness of the thin film. Because of the short vertical channel, the TFT dimensions are much smaller than that of a conventional planar TFT, and the source-drain voltage can also be reduced. A vertical channel is also immune to the bending of the substrate, which induces compressive or tensile stress that deteriorates the electrical characteristics or induces cracks in a conventional planar-channel TFT.

**Figure 1:**
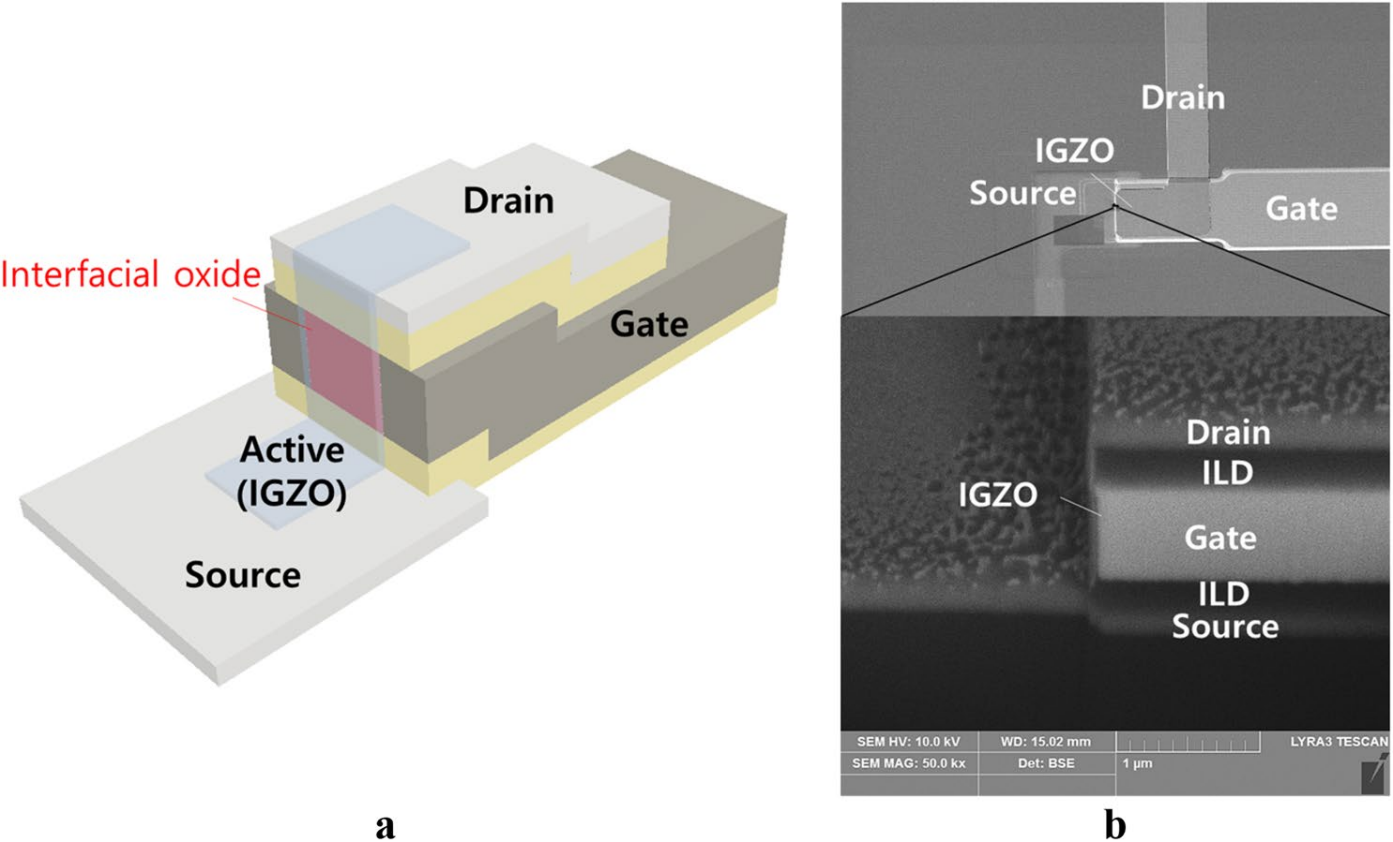
(**a**) Structure of the developed vertical-structure TFT. (**b**) SEM images of plane view and cross-sectional structure.

In general, the channel length of a conventional oxide VTFT is defined by the thickness of the spacer layer which is the insulator inserted between the source and drain electrodes^15,16^. Several types of insulators such as PECVD SiO_2_^15–20^, organic material^21^ and silicon nitride^22,23^ were applied for the spacer in conventional oxide VTFTs. Those VTFTs by spacer need deposition of gate insulator after pattern of active layer on the side wall of the spacer. However, in this study, the spacer was replaced by the gate metal, and the interfacial oxidation between the gate metal and the IGZO layer was developed for the gate insulator, as shown in Fig. 1a. After deposition of the IGZO on the sidewall of the metal, it was annealed under oxygen atmosphere. It was reported that oxygen supply from oxygen atmosphere is an oxygen source and exchanged into the IGZO layer and migrate even at 350 °C^24^. Therefore, the oxygen atmosphere is important for the oxidation of the gate metal at the interface with IGZO. We verified the effect of the oxygen atmosphere by comparison to the nitrogen atmosphere during annealing. The other important one is reactivity of an oxygen with a metal atom. The lower Gibbs free energy for oxide formation is important and we compared the interfacial oxidation for two different Gibbs free energy metals, Mo and Ta. Mo was tested in terms of better adhesion than Ta which has weak adhesion and easily peel off during process. The formation of an interfacial oxide film was observed through high resolution transmission electron microscopy (HR-TEM) and energy dispersive X-ray spectroscopy (EDS). We report the results of a VTFT using an interfacial oxide as a gate insulator. The advantages compared to the conventional IGZO VTFT are removal of a gate insulator deposition process, and thin insulator for low voltage operation. However, the control of the etched side wall is difficult to get smooth surface for low scattering of the carrier to reduce the scattering of carriers.

## Results and discussion

The structure of the vertical TFT with interfacial oxidation is shown in Fig. 1a. The source electrode at the bottom was made of indium tin oxide (ITO) to prevent the formation of a metal oxide insulator between the source and IGZO layer during the interfacial oxidation. The first interlayer dielectric (ILD) of SiO_2_ and metal gate electrode were deposited by successive sputtering, and then dry etched at one time with one photo mask. A second ILD of SiO_2_ and drain electrode of ITO were formed by a lift-off process, and then the gate and first ILD were dry etched by self-alignment with the drain pattern to form the vertical side wall.

After deposition of the IGZO by radio frequency (RF) magnetron sputtering, it was annealed under the oxygen atmosphere for the interfacial oxide on the side walls. As a result, the gate insulator was formed at the interface between the metal gate and IGZO active layer. The interfacial oxidation was done before the etching of the IGZO for the active layer to avoid the thick oxidation of the metal electrode which occur if the metal was not covered by the IGZO layer.

Figure 1b shows secondary electron microscope (SEM) images of the plane view and cross-sectional structure of the fabricated TFT. The vertical channel is shown between the bottom ITO electrode and top ITO electrode on the vertical side wall of the 600 nm thick gate electrode. An offset region was formed by 250 nm thick ILD layers while 600 nm channel length was defined by the 600 nm thick gate metal.

**Figure 2:**
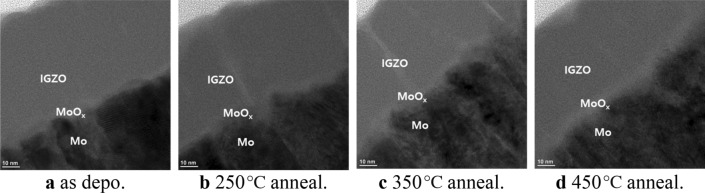
HR-TEM of Mo-IGZO interface for various annealing temperatures.

The interfacial oxidation between the channel IGZO and gate electrode was examined using test samples with the IGZO/metal structure on a glass. The samples were annealed at various temperatures of 250 to 450 °C under oxygen atmosphere. Mo was considered for better adhesion than Ta which peel off easily during process especially under thermal stress. And the effect of the Gibbs free energy was compared with that of Ta. Figure 2 shows the HR-TEM result of the Mo-IGZO interface for various annealing temperatures, including the transition region between the IGZO and Mo. The EDS analysis shows that Mo atomic ratio decreases gradually from Mo to IGZO, and oxygen atomic ratio decreases from IGZO to Mo. During the annealing, the Mo atoms react with oxygen atoms of the IGZO layer, which requires oxygen diffusion to the IGZO-metal interface after bond breaking from the metal ion. The interstitial oxygen could also diffuse to the IGZO-metal interface to bond with Mo atoms. Oxygen supply from the oxygen atmosphere can be an oxygen source also and the migration of oxygen even at 350 °C have been reported^17^.

Figure 2 shows that the width of the MoO_x_ region increases when increasing the annealing temperatures from 250 to 450 °C. At low temperatures, the interstitial oxygen can contribute to the oxidation by temperature-dependent diffusion to the interface because oxygen bond breaking from metal ions is difficult at low temperatures. The bond breaking requires much higher temperatures, so interstitial oxygen plays an important role in forming metal oxide at low temperatures. This means the oxygen ambient is very important because the oxygen continuously diffuses into the IGZO layer and migrates to the metal-IGZO interface to form metal oxide. Therefore, the adsorption and diffusion of the ambient oxygen into the IGZO layer is important for the interfacial oxidation.

**Figure 3:**
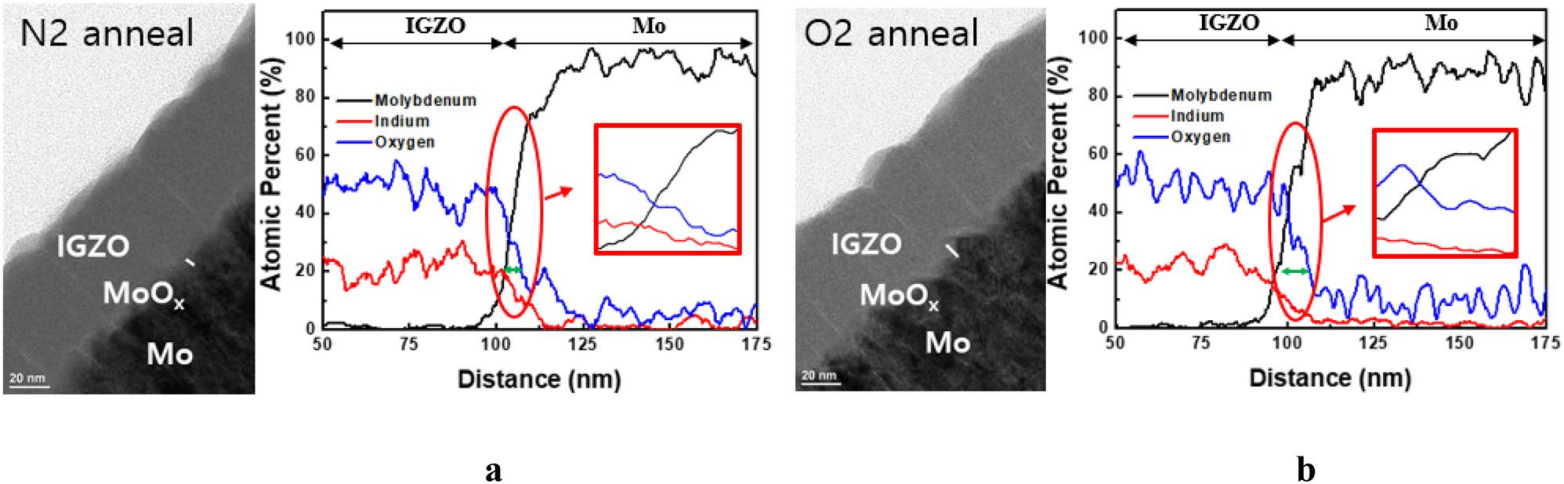
HR-TEM images for Mo-IGZO interface annealed at 350 °C, under the nitrogen atmosphere (**a**), and oxygen atmosphere (**b**).

To verify the effect of the oxygen atmosphere, the interfacial oxidation was examined for both oxygen and nitrogen atmospheres. Figure 3a shows HR-TEM results of the interfacial oxidation under nitrogen atmosphere, and Fig. 3b shows the result under the oxygen atmosphere. The ambient oxygen diffused into the IGZO layer by surface reaction and migrated to the IGZO layer to form metal oxide, so the oxygen atmosphere is very useful for obtaining a thicker interfacial metal-oxide for the gate insulator. The atomic percentage from the EDS analysis shows a larger oxygen ratio at the interface for the sample annealed under oxygen than that annealed under nitrogen.

The reaction of oxygen at the metal surface is important for a thicker metal-oxide at low temperatures below 400 °C For a thicker metal oxide, Ta was used instead of Mo because of its lower Gibbs free energy than that of Mo. The Gibbs free energies for MoO_2_ and Ta_2_O_5_ are − 533.0 and − 1911.2 kJ/mol, respectively.

**Figure 4:**
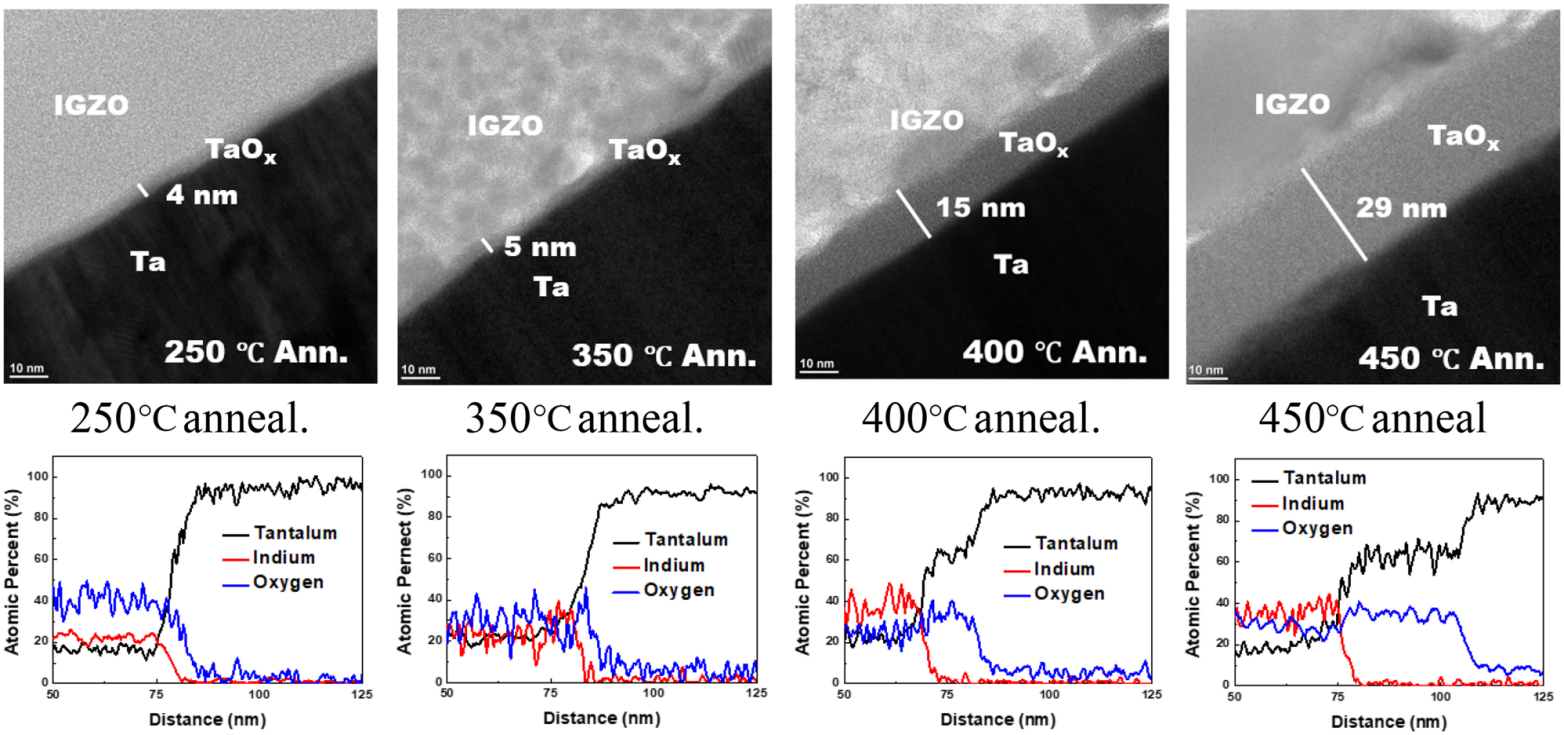
HR-TEM images and EDS of the interface between the gate metal and IGZO for various annealing temperatures.

Figure 4 shows the HR-TEM images of interfaces between Ta and IGZO layers for various annealing temperatures under oxygen atmosphere. The thickness of the interfacial oxide was expected to be larger in Ta than Mo due to the lower Gibbs free energy. When increasing the annealing temperatures, the interfacial oxide becomes clearer, and the thickness increases. The EDS result also matches with the HR-TEM images and shows a change of the Ta and oxygen atoms at the interface. When annealed at 450 °C anneal, the thickness of tantalum oxide was 29 nm.

**Figure 5:**
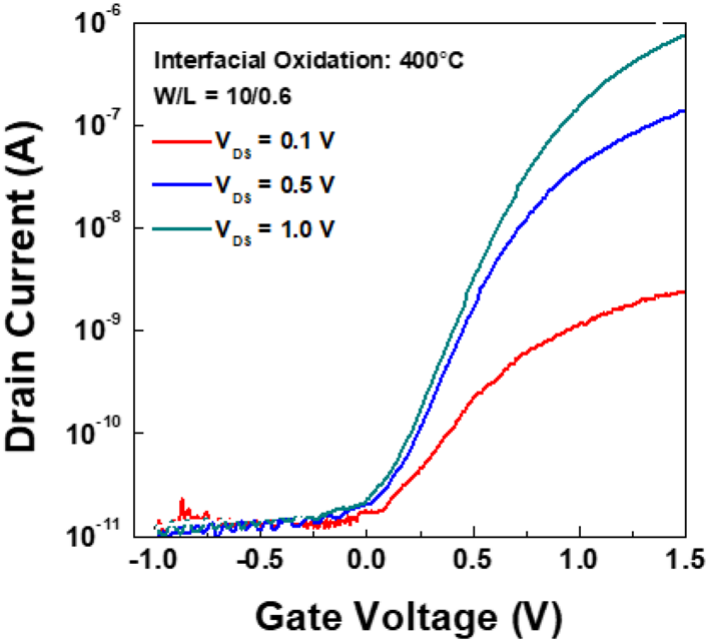
Transfer curves of the vertical TFT with Ta-IGZO interfacial oxidation for the gate insulator.

Since the Ta gives thicker oxide than Mo, we choose Ta for the gate material. To avoid peel off, we keep the low thermal stress during the process. The VTFT was fabricated with interfacial oxidation between IGZO and Ta gate metal as shown in Fig. 1. The interfacial oxidation temperature was 400 °C. The transfer curves of the fabricated VTFT is shown in Fig. 5. The source-drain voltage was varied from 0.1 V to 1.0 V and the gate sweep was from − 1.0 V to 1.5 V for low-voltage operation. The thickness of the gate insulator was 15 nm, as shown in Fig. 4. The channel length was defined by the thickness of the gate metal, which was 600 nm. The channel width was defined by the photolithography and was 10 µm. The threshold voltage of the VTFT was 0.3 V, and the subthreshold slope was 0.2 V/dec. The mobility extracted from saturation region was 0.29 cm^2^/Vs with the gate insulator thickness of 15 nm as shown in Fig. 4. Conventional oxide VTFTs show better performances of field effect mobilities larger than 5.7 cm^2^/Vs^16,21^. There are several factors to decrease the field effect mobility. Relatively large offset region by interlayer dielectric can give large contact resistance which decrease the on currents. The thicknesses of the insulators are 250 nm on both the source and gate electrodes, which means the total offset length is 500 nm. This length is comparable to the channel length of 600 nm. The vertical side wall is formed by dry etching which remain rough surface wall. The rough surface by dry etching can increase the scattering of carrier. The gate metal diffusion into a IGZO also should be considered in terms of electrical characteristics. The effects of metal diffusion into a IGZO layer are reported that it acts as a carrier suppressor which reduces the conductivity^25^. Therefore, the diffusion of the gate metal into the IGZO also should be investigated further.

The offset is a disadvantage which should be solved to get high performance of the TFT. Since the gate metal separated source drain structure is applicable to vertical NAND flash memory, reduction of the offset effect is important. Large offset can be reduced using by decreasing the thickness of interlayer dielectric. The doping effect on the offset region is useful and can be achieved by the proper choice of the insulator which can impinge the doping elements into the IGZO layer.

**Figure 6:**
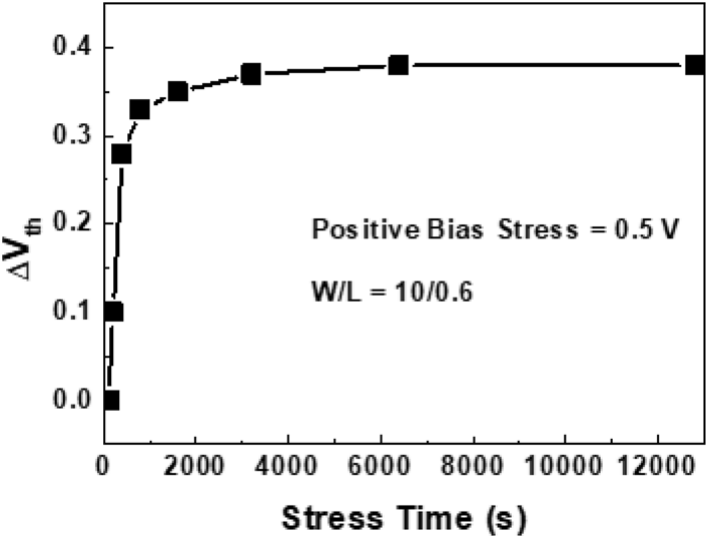
Threshold voltage shift during positive gate bias stress.

The stability was tested for positive gate bias stress. Figure 6 shows the threshold voltage changes during the positive gate bias stress of 0.5 V. The threshold voltage increased with the stress time. The shift of the threshold voltage after 800 s was about 0.9 V, which is rather rapid compared to the operation voltage of 0.5 V. The stability should be improved, and the next task will be to get the stable devices under the bias stresses.

**Figure 7:**
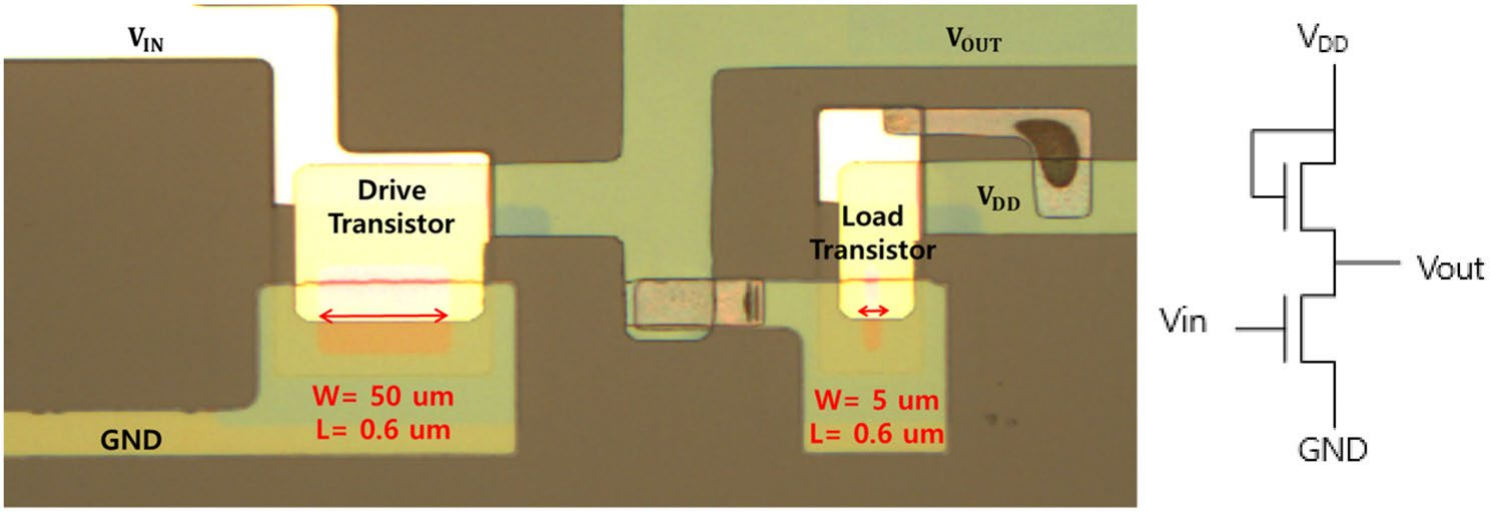
Microscopic image of the fabricated inverter with interfacial oxidation VTFT.

For low voltage circuit applications, an inverter with the VTFT by interfacial oxidation was evaluated, as shown in Fig. 7. The channel lengths were kept the same for both the drive and load transistors, while the widths of the channel were 50 and 5 µm for the drive and load transistors, respectively.

**Figure 8:**
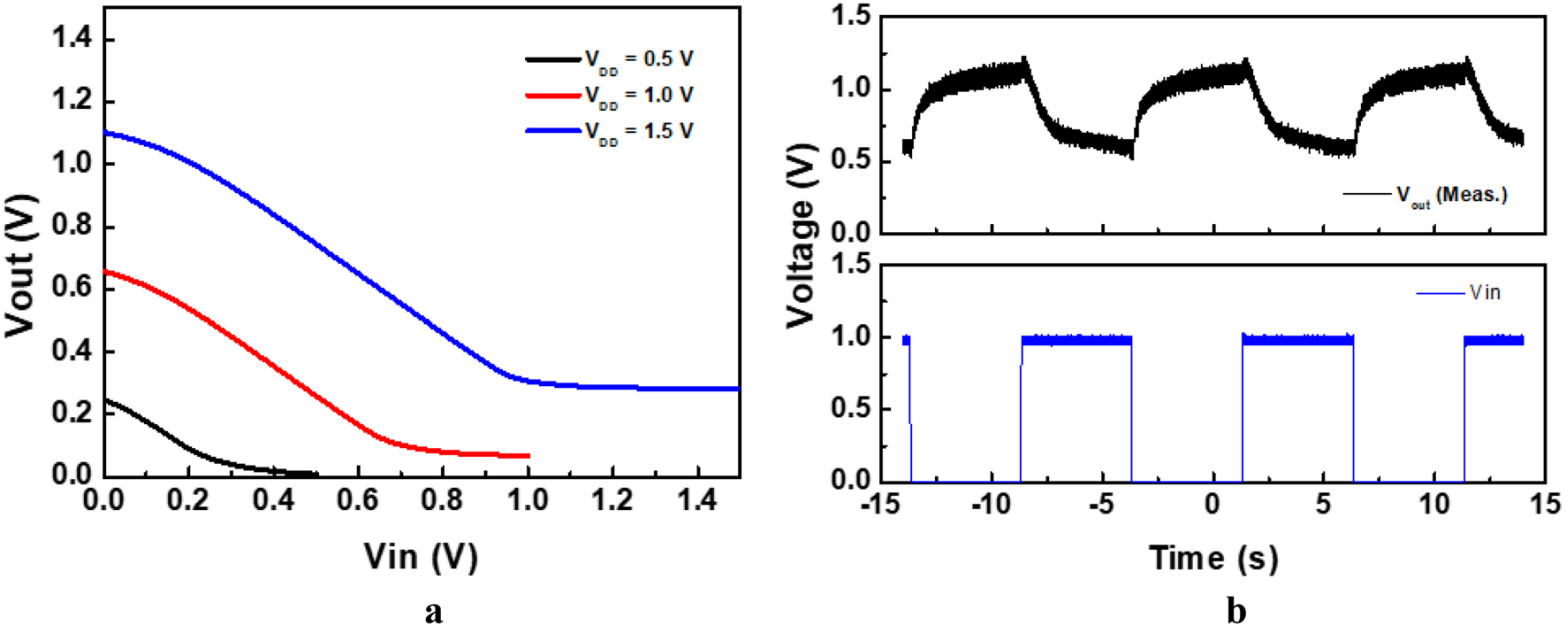
(**a**) Voltage transfer curve of the inverter with interfacial oxidation VTFTs, (**b**) AC characteristics of the inverter.

The voltage transfer curve and AC characteristics of the inverter are shown in Fig. 8a and b, respectively. The voltage transfer curve shows decreased output voltages for the increased input voltages, which is a typical inverter characteristic. The output pulse is inverted in comparison to the input pulse, which shows the successful operation of the inverter.

The gain obtained from Fig. 8**a** was 0.92. High gain is important in aspect of the circuit performance. Good policy for high gain with low power consumption is complementary circuit with N and P channel transistors such as IGZO-SnO channel combination or IGZO-Poly Si channels which showed gain 63 at 1.5 V and 120 at 2 V, respectively^26,27^. However, in this study the inverter is composed of N-channel only and has low mobility, which results in low gain of 0.93.

**Figure 9:**
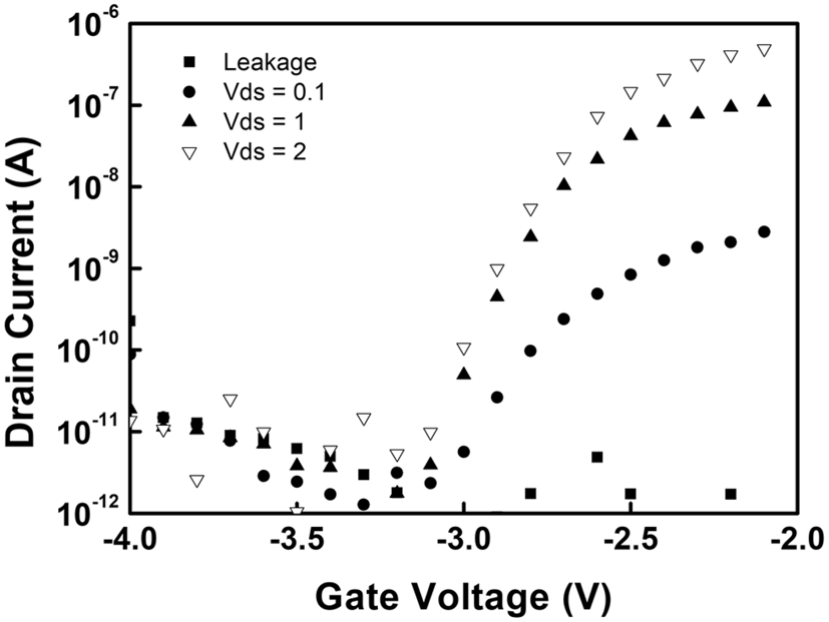
Transfer characteristics of interfacial oxidized gate insulator IGZO VTFT.

For the purpose of the improvement of the mobility we tested In rich IGZO layer with the target of mole ratio In:Ga:Zn = 2:1:2. Usually In rich IGZO give higher mobility and better slope. We applied the In rich IGZO in this structure. The transfer characteristics with In rich IGZO is shown in Fig. 9.

Currents for Vds = 0.0, 0.1, 1.0 and 2.0 V are shown. Leakage currents are for the Vds = 0 V. Negative shift was observed. The process should be optimized in two aspects, one is the IGZO layer optimization for the high mobility and the other is interfacial oxide for the low leakage current and less defects. The interfacial oxidized gate insulator is the first trial and need further research to improve the characteristics of it. One drawback of the developed VTFT is the large offset length compared to the channel length which also results in a large offset AC output low. Further development on a short offset or doped offset is required.

## Conclusion

The interfacial oxide between gate metal and an IGZO layer was studied and used as a gate insulator in a vertical TFT. The interfacial oxidation was observed after thermal annealing under oxygen or nitrogen atmosphere for Mo metal. Ta meal was also investigated for various annealing conditions. Mo was tested in terms of better adhesion than Ta, however, Ta was used in interfacial oxidation of IGZO VTFT because it showed a much thicker interfacial oxide due to lower Gibbs free energy of tantalum oxide than that of Mo. A 15 nm Ta interfacial oxide layer was obtained after 1 h annealing at 400 °C under oxygen atmosphere. In the proposed structure, spacer insulator that defines the channel length of the vertical TFT was replaced by a metal gate, and the deposition process of the gate insulator was skipped. The interfacial oxide between the gate metal and IGZO played a role of a gate insulator. The very thin interfacial oxide layer of 15 nm made it possible for the TFT to operate at low voltages less than 1 V.

## Methods

The process for the developed VTFT is as follows. A 150 nm ITO source layer was deposited via DC sputtering under an Ar atmosphere and patterned using a photolithography. A 250 nm SiO_2_ inter-layer dielectric (ILD) was deposited via RF sputtering, and the gate metal of Ta was deposited with a thickness of 600 nm by DC magnetron sputtering. The ILD and gate were patterned at once using photolithography and dry etching.

A 250 nm SiO_2_ ILD and 150 nm ITO drain were deposited under the same conditions and patterned by a lift-off method. The gate and ILD were dry etched to form the vertical side channel. A 40 nm IGZO (In:Ga:Zn = 1:1:1 at.%) active layer was deposited by RF magnetron sputtering. The deposition pressure was 5 mTorr, and the Ar and O_2_ gas flows were fixed at 25 and 7.5 sccm, respectively. Annealing was done at 400 °C for 1 h in a furnace under oxygen atmosphere to obtain the gate insulator by interfacial oxidation between the gate and IGZO. The IGZO was then patterned using a buffer oxide etchant diluted with deionized water at ratio of 500:1.For the TEM observation, samples of the glass/Mo/IGZO and glass/Ta/IGZO structure were fabricated with the same conditions as the TFT process. The samples were annealed in a furnace at various temperatures under oxygen or nitrogen atmosphere.
